# Large granular lymphocytosis with severe neutropenia following ipilimumab therapy for metastatic melanoma

**DOI:** 10.1186/2162-3619-1-3

**Published:** 2012-03-26

**Authors:** Guoqing Wei, Uzoma Nwakuche, Gustavo Cadavid, Asim Ajaz, Karen Seiter, Delong Liu

**Affiliations:** 1 Division of Hematology and Oncology, New York Medical College and Westchester Medical Center, Valhalla, NY 10595, USA; 2 Bone Marrow Transplantation Center, the First Affiliated Hospital, Zhejiang University School of Medicine, Hangzhou 310003, China

## 

To the editor

Cytotoxic T-lymphocyte antigen-4 (CTLA-4), a molecule present on activated T cells, is a homologue of CD28 that inhibits B7 co-stimulatory molecules expressed on mature antigen presenting cells. Ipilimumab is a fully humanized monoclonal antibody against CTLA-4 [1]. Ipilimumab is FDA approved in the United States for treatment of unresectable or metastatic melanoma [1,2]. We report the first case of large granular lymphocytosis with severe neutropenia following ipilimumab therapy.

A 74 year old female presented with relapsed melanoma in the left lower extremity in Sept 2009. She was treated with temozolomide. In July 2010, she developed metastatic melanoma. She was enrolled onto a clinical trial and treated with ipilimumab on Sept 16, 2010 and October 7, 2010 at 3 mg/kg (232 mg per treatment) q3wks. Her baseline CBC was normal. At the start of the third dose, there was a significant decline in the patient's WBC count. Therefore ipilimumab was stopped. The patient was admitted on November 2, 2010 with fever, malaise and generalized weakness. She was noted to have Hgb 6.2 G/dL, WBC 1.3 × 10^6 ^/L with no neutrophils, and platelet count of 158 × 10^6 ^/L. The patient had a bone marrow evaluation which showed mildly hypercellular cellular marrow with marked myeloid hypoplasia/aplasia, relative erythroid hyperplasia and mild megakaryocytic atypia. Karyotype analysis demonstrated 46 XX, but FISH analysis showed a 5q31 deletion with EGR1 gene deletion in 14% of the cells. Flow cytometry study showed 46% T-cells with 20% large granular lymphocytes and 12% NK cells. A T-cell receptor gene study by Genzyme showed clonal T-cell receptor gamma gene rearrangement. These findings were consistent with myelodysplasia and large granular lymphocytosis with severe neutropenia. In addition to broad-spectrum antibiotics and G-CSF, the patient was given IVIG without significant response. The CBC on Nov 17 was WBC 0.8, Hgb 8.9, and Platelets 271. CT scans revealed bilateral loculated pleural effusion for which she had thoracentesis. The patient was started on treatment on Nov 18, 2010 with IV methylprednisolone 1 mg/kg q12h, and equine ATG at 15 mg/kg daily × 4 days, as well as cyclosporine 2.5 mg/kg twice daily. G-CSF and antibiotics were continued. The WBC rose to 2.6 the day after ATG was completed (Table 1). Two days later, her WBC was 15.3. G-CSF was stopped. Her general condition improved gradually. She was discharged 11 days later. She remained on cyclosporine and low dose prednisone. Three months later, she remained only on low dose prednisone. Her CBC showed WBC 12.1, Hgb 11.9, and Platelets 259 at last follow-up visit on Feb 23, 2011.

**Table 1:** Complete Blood Counts of the Patient

Date	**WBC (×10**^**9**^)**/L)**	ANC (×10^9^)/L)	Hgb (×g/L)	Plt (×10^9^)/L)
11/17/2010	0.8		8.9	271
11/18/2010	0.9		9.0	280
11/19/2010	0.1		8.3	142
11/20/2010	0.3		8.1	90
11/21/2010	0.7		8.8	92
11/22/2010	2.6	1.2	8.5	60
11/23/2010	15.3	6.7	8.7	70
11/24/2010	30.3	19.4	8.2	73
11/25/2010	35.9		7.8	55
11/27/2010	28.4	25.8	9.7	43
11/28/2010	23.6	20.5	10.1	41
11/30/2010	15.0	13.8	8.5	77
12/1/2010	12.4		8.8	82
12/2/2010	8.8	7.2	7.9	61
12/17/2010	2.7	1.6	9.8	59
12/21/2010	7.0	5.3	10.8	157
2/17/2011	6.5	5.6	10.1	194
2/23/2011	12.1	10.3	11.9	259

Abbreviations: WBC: white blood cell; ANC: absolute neutrophils count; Hgb: hemoglobin; Plt: platelet.

The most common adverse events associated with ipilimumab are immune-related, including enterocolitis, hepatitis, dermatitis and hypophysitis. Severe hematological toxicity is rare. One case of severe autoimmune-related neutropenia was reported and the patient responded rapidly to IVIG infusion, but not to steroids [3]. This case of large granular lymphocytosis with severe neutropenia did not respond to IVIG and steroids, but had a rapid response to ATG, steroids, and cyclosporine immunosuppressive therapy. It is unclear whether the myelodysplasia findings on the bone marrow biopsy were related to the ipilimumab therapy or to previous chemotherapy with temozolomide.

## Competing interests

The authors declare that they have no competing interests.

## Authors' contributions

GW and UN contributed equally to the study. All authors participated in concept design, data collection and analysis, drafting and critically revising the manuscript. All authors read and approved the final manuscript.
